# Increased Levels of C5a in Gingival Crevicular Fluid and Saliva of Patients with Periodontal Disease

**DOI:** 10.3390/pathogens11090983

**Published:** 2022-08-29

**Authors:** Simran Preet Bhalla, Ann Maria Shaju, Carlos Marcelo da Silva Figueredo, Leticia Algarves Miranda

**Affiliations:** 1 Discipline of Periodontics, UWA Dental School, Nedlands, WA 6009, Australia; 2 School of Medicine and Dentistry, Griffith University, Nathan, QLD 4111, Australia; 3 Division of Oral Diseases, Department of Dental Medicine, Karolinska Institutet, 141 04 Huddinge, Sweden

**Keywords:** C5a, periodontitis, complement, gingival crevicular fluid, saliva

## Abstract

C5a is a powerful complement effector molecule that is considered to be an important proinflammatory mediator in several systemic chronic inflammatory diseases. However, its levels in periodontal diseases are yet to be assessed. We aimed to analyse the secretion of C5a in gingival crevicular fluid (GCF) and saliva of patients with periodontal disease. Twenty-eight patients diagnosed with stage 3–4 periodontitis and 16 periodontally healthy subjects participated in this study. GCF was collected from sites with the deepest probing depth of each patient, and volume was measured using a Periotron 8000^®^. One mL of unstimulated saliva was also collected. Samples were analysed using a commercially available ELISA kit. The data were analysed using the Mann–Whitney U test, Pearson’s bivariate testing, and receiver operating characteristic curve. C5a was present in GCF from patients with periodontitis (1.06 ± 0.25 ng/mL) whilst it was undetected in controls. Saliva concentration was also significantly higher in periodontitis (1.82 ± 2.31 ng/mL) than controls (0.60 ± 0.72 ng/mL, *p* = 0.006). C5a levels were more pronounced in periodontitis in both oral fluids assessed by the present pilot study. These results suggest that the more pronounced levels of C5a in oral fluids from periodontitis patients indicate a potential role of this molecule in this disease pathogenesis, deserving to be better explored in subsequent studies.

## 1. Introduction

Periodontitis is a chronic inflammatory disease driven by the dysbiosis of the oral microbiome and associated dysregulation of the host immune response, resulting in the loss of tooth-supporting connective tissues [1,2,3]. Dysbiosis in the sulcular environment initiates the disease, but its progression is mainly heavily mediated by the host’s innate and acquired immune responses. The disease can progress exponentially with the destruction of the periodontal ligament, significant bone loss and potentially, tooth loss [1,3].

The complement system is one of the significant links between infection and inflammation in periodontal disease. It plays an important role in host defense by constituting a non-cellular system for protection against pathogens via a protease cascade, leading to the activation of several effector molecules [1,3,4]. C5a, the most potent effector molecule in the complement cascade [5,6], is a peptide anaphylatoxin that binds to C5a receptors (C5aR) present on many immune cells, including granulocytes, monocytes, macrophages, dendritic cells, mast cells and T-cells [5,6]. It enters the circulation and induces, as well as perpetuates, several systemic effects [5,6,7]. These include the release of lysosomal enzymes and reactive oxygen species, directed migration of white blood cells to inflamed sites, smooth muscle contraction, increased vascular permeability and bone turnover. Further, it is involved in bone resorption by inducing osteoclast activity, a hallmark feature in periodontitis [8,9].

C5a has been suggested to have a critical role in the aetiopathogenesis of various diseases, such as rheumatoid arthritis (R.A.), systemic lupus erythematosus, atherosclerosis, and Alzheimer’s disease [10,11,12,13,14,15]. C5aR antagonists have been successful in reducing inflammation in rodent models for inflammatory bowel disease, Huntington’s disease and Alzheimer’s disease [16,17,18]. Recently, a study in rodent models identified a significant concentration of C5a in the synovial fluid of mice with R.A. infected with *P. gingivalis*. Therefore, the authors hypothesised that *P. gingivalis* infection and likely periodontitis are involved in the progression of R.A. by elevating C5a levels [9].

The gingival crevicular fluid (GCF) comprises almost 70% of complement cascade components [19]. Elevated C3 levels have been identified in oral fluids, such as GCF [20] and saliva [21] of gingivitis and periodontitis patients. However, little is known about the C5a levels in different human periodontal conditions, and no study has isolated C5a fragments in oral fluids.

Herein, we hypothesised that C5a could be detected in oral fluids collected from individuals with periodontal disease.

## 2. Methods

### 2.1. Study Design and Population

This cross-sectional pilot study was conducted at the Oral Health Centre of Western Australia (OHCWA) in partnership with the University of Western Australia (UWA) Dental School, Periodontology Department. Sixteen controls and 28 periodontitis patients were selected and diagnosed, as per the 2017 Classification of Periodontal and Peri-implant Diseases and Conditions, as periodontally healthy or having periodontitis stages 3–4. Ethics approval for this study was attained from the University of Western Australia Human Ethics Committee ROAP 2019/RA/4/20/6063, and all patients had given written consent to participate.

Patients were categorised as periodontitis (stage 3–4; grade A–C) or clinical periodontal health. Inclusion criteria for those diagnosed with periodontitis were the following: (1) plaque score greater than or equal to 20% of sites; (2) bleeding on probing at greater than 10% of sites; (3) with at least two non-adjacent sites with proximal attachment loss greater or equal to 5 mm; (4) probing depths greater than 4 mm, and (5) indicating radiographic bone loss.

Inclusion criteria for those categorised as periodontal health include (1) plaque score less than 20%, (2) bleeding on probing less than or equal to 10% of the sites, (3) no interproximal attachment loss. (4) probing depths equal to or less than 3 mm.

Patients with acute necrotising periodontal disease, systemic diseases affecting periodontal supporting tissues, women who are pregnant or lactating, patients who underwent chemotherapy or radiotherapy within the last 6 months, that prescribed antimicrobial therapy within the last 6 months and smokers were excluded from our study.

For all patients, medical history, dental history and clinical periodontal analysis from the last exam performed by the patient’s clinician were recorded. The periodontal recording included plaque score (P.S.), bleeding on probing (BOP), clinical attachment loss measurements (CAL) and probing depth (P.D.).

### 2.2. Samples

Samples of GCF were collected from the two deepest probing depths per patient using an Oraflow^®^ Periopaper strip (New York, NY, USA). The selected sites had supragingival plaque removal and were isolated with cotton rolls. A single Periopaper strip was inserted into the pocket until slight resistance was felt. It was removed after 30 s and transferred onto a Periotron 8000 for electronic volume quantification. Then, it was placed into 100 µL of phosphate-buffered saline with 0.05% Tween-20 solution with constant agitation to elute the GCF sample. The eppendorf tube was centrifuged at 8000× *g* for 10 min at 4 °C, then stored at −80 °C. Strips contaminated with blood or suppuration were discarded.

For saliva sample collection, participants were asked to refrain from eating or drinking for 1 h before collection. During collection, the first 30 s of saliva were discarded, and then they were asked to expectorate 1 mL of unstimulated whole saliva (passive drooling) into sterile universal tubes. Tubes were placed on ice, and a protease inhibitor cocktail (DMSO solution) was added. The supernatant was aliquoted, and tubes were sorted at −80 °C. Within 3 h of collection, samples were centrifuged at 3000× *g* for 5 min at room temperature.

### 2.3. Analysis of C5a

A commercially available human C5a ELISA kit was used (ELISA, BD OptEIA, Loughborough, UK). Standards and samples were prepared following manufacturer instructions and in duplicate. Samples were diluted at 1:2. In brief, 100 µL of liquid in each well was incubated for 2 h at room temperature and then washed five times. 100 µL of the working detector was added to the wells, followed by 1-h incubation, and washed seven times; 100 µL of TMB substrate reagent and subsequently, 50 µL stop solution were added. The plates were read through a microplate reader at 450 nm within 30 min. A standard curve was prepared for each plate. The kit minimum detection amount of C5a was 0.047 ng/mL.

### 2.4. Statistical Analysis

MS Excel and IBM SPSS software (Chicago, IL, USA) were used for statistical analysis. All data were assessed for normality using the Shapiro–Will test. As the data was not normally distributed, differences between the control and periodontitis samples were analysed using the Mann–Whitney U test. A *p*-value < 0.05 was statistically significant. To measure the discriminative ability of our testing, the area under ROC curves was calculated. Further correlation testing was conducted, including Pearson’s bivariate testing and automated linear modelling.

## 3. Results

### 3.1. Subject Demographics

The demographic and clinical details of the participants are outlined in Table 1.

### 3.2. C5a presence in GCF and Saliva

**Figure 1:**
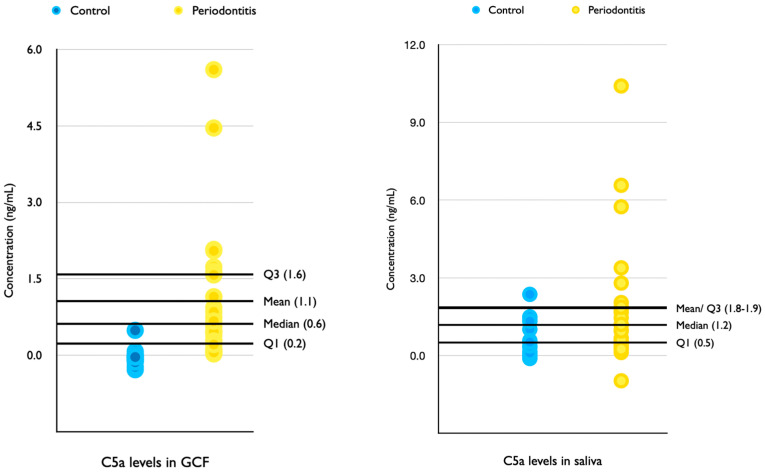
Scatter plots of C5a concentrations in GCF and Saliva of control and periodontitis patients.

**Figure 2:**
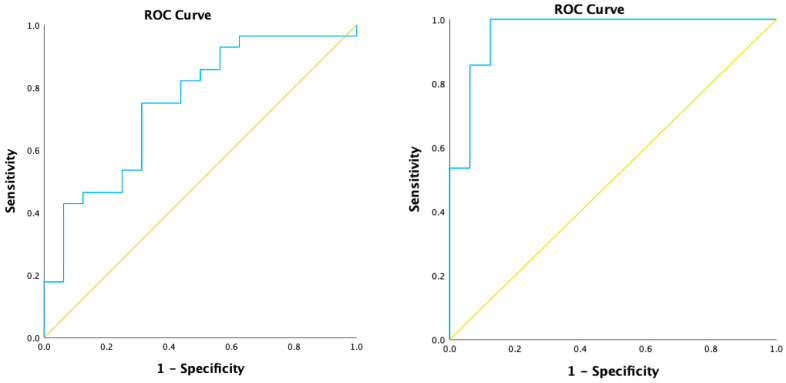
ROC curves for GCF and Saliva.

All GCF and saliva samples were included in the analysis. The mean concentration of C5a determined in the oral fluids is shown in Table 2 and Figure 1. In brief, C5a levels were present in statistically significant (*p* < 0.05) amounts in periodontitis patients compared to controls. Detectable concentrations of C5a (1.06 ng/mL ± 0.245) were observed in all GCF samples from periodontitis patients. These values were lower than those observed in the saliva of periodontitis patients (1.82 ng/mL ± 0.603). Within controls, C5a was undetectable and in very low quantities in saliva (0.603 ng/mL). The area under the curve was high for GCF and saliva ROC analyses, as per Table 2 and Figure 2.

Pearson’s bivariate testing underlined critical correlations in our data, as per Table 3. Significant correlations existed between C5a in saliva and GCF as well as with the mean percentage of probing depths ≥4 mm and total bleeding on probing.

## 4. Discussion

This pilot study aimed to explore the presence of C5a in patients with periodontitis oral fluids. This powerful complement protein has been associated with inflammation in the human body, which is increasingly linked to many immune-mediated diseases. To our knowledge, C5a has not explicitly been identified in human saliva and GCF samples of periodontally healthy and diseased subjects. If C5a is consistently identified and in higher levels in diseased states compared to health, this knowledge could lead to other approaches to determine the involvement of C5a in disease pathogenesis.

Even though C5a is considered the most powerful complement effector protein, little has been described about its involvement in periodontal diseases. This study shows that C5a was present in significantly higher concentrations in periodontitis samples than in healthy controls in both oral fluids. This finding aligns with a study done in SKG mice by Munenaga et al. in 2018 [9]. Increased levels of C5a (19.4 ± 6.1 ng/mL) were isolated in the synovial fluids of *P. gingivalis*-infected mice. The role of the microbial biofilm in initiating periodontal disease is well appreciated. *P. gingivalis* is a keystone pathogen and heavily contributes to the entire biofilm’s survival by interacting with various host receptors to manipulate the immune defence. Significantly, it also enhances local C5a production as a survival advantage. This is done by potent virulence factors called gingipains that convert C5 to C5a [22].

In addition, a higher concentration of C5a was observed in saliva samples compared to GCF. Salivary levels of another complement factor, C3 and its split factor, C3c, have been compared in saliva of periodontitis patients, before and after periodontal treatment [20]. The results of Grande et al. [21] corroborate ours as they found increased levels of C3/C3c in periodontitis patients when compared to healthy controls. Both these fluids represent different environments of the oral cavity. GCF represents the local environment of a periodontal sulcus/pocket, which can be the reservoir of cytokines during inflammatory states. This was underlined by our study, which was unable to detect C5a in the GCF of healthy controls. In comparison, saliva is a culmination of respiratory, digestive, and oral components, reflecting a more general response. Potentially, C5a detected in control saliva samples suggests inflammation in the oral cavity external to the periodontium, even though a stringent selection criterion for systemic diseases affecting the periodontium was employed.

Our study can form a framework for investigating a more significant magnitude to scrutinise the relationship between C5a and periodontal disease. Confirmation of a direct relationship between local C5a levels and the severity of periodontitis can be an essential finding. In addition to contributing to the current understanding of periodontal disease pathogenesis, the results of this study can be adapted to diagnostic and treatment decisions. This is supported by the high sensitivity and specificity shown in the ROC curves.

Notwithstanding, the present investigation had several limitations. Primarily, the small sample size hinders the significance of our conclusion. To properly establish C5a as a potential periodontitis biomarker, more extensive and varied patient groups are required. In addition, this is a cross-sectional study based on a convenience university-based sample (selection bias). Comparing C5a levels before and after periodontal treatment could also provide further information on the role of C5a in periodontitis. By definition, C5a represents inflammation. In the oral cavity, this is not only limited to periodontal disease. Several contributors have been attempted to be controlled. However, the risk of confounding and potentially undiagnosed variables remains ingrained in our study, especially in the saliva analysis.

## 5. Conclusions

C5a levels were more pronounced in periodontitis in both oral fluids assessed by the present pilot study. These results suggest that the role of C5a in periodontitis pathogenesis deserves to be better explored in subsequent studies to assess its potential as a biomarker.

## Figures and Tables

**Table 1:** Demographic and clinical parameters of the periodontitis and control groups.

	Periodontitis *n* = 28	Control *n* = 16
Age (min-max)	19–88	27–92
Bleeding on probing (%)	48.3 ± 26.6	6.4 ± 3.4
Probing depths > 4 mm (%)	57.9 ± 23.9	8.0 ± 5.9
Mean probing depth (mm ± SD)	6.1 ± 1.6	2.3 ± 1.2
The mean volume of GCF collected (µL ± SD)	0.9 ± 0.3	0.6 ± 0.5

**Table 2:** Mean concentration of C5a in saliva and GCF of periodontitis patients and controls.

C5a	*n*	Mean (ng/mL)	Std. Error Mean	Area Under Curve (ROC Curve)
GCF *Periodontitis*	28	1.06 *	0.245	0.748
GCF *Control*	16	Undetectable	Undetectable	--
Saliva *Periodontitis*	28	1.82 ^#^	0.437	0.962
Saliva *Control*	16	0.603	0.180	--

* Significance at *p* < 0.05, *p*-value < 0.00001, ^#^ Significance at *p* < 0.05, *p*-value = 0.00694.

**Table 3:** Pearson’s correlations of C5a in GCF and saliva and clinical parameters.

	C5a in Saliva	Probing Depths ≥ 4 mm (%)	Bleeding on Probing (%)
C5a in GCF	0.531	0.455	0.543
Probing depths ≥ 4 mm (%)	0.551	--	--
Bleeding on probing (%)	0.211	--	--

0.4–0.5—significant at 0.05; ≥0.5 significant at 0.01.

## Data Availability

Data available upon request.
